# External validation of a time-lapse model; a retrospective study comparing embryo evaluation using a morphokinetic model to standard morphology with live birth as endpoint

**DOI:** 10.5935/1518-0557.20180041

**Published:** 2018

**Authors:** Emma Adolfsson, Sandra Porath, Anna Nowosad Andershed

**Affiliations:** 1 Örebro University Hospital. Department of Laboratory Medicine. Örebro, Sweden; 2 Örebro University Hospital. Fertility Unit, Department of Women Health. Örebro, Sweden

**Keywords:** Algorithm, embryo selection, time-lapse image, embryo evaluation, morphokinetics

## Abstract

**Objective:**

To validate a morphokinetic implantation model developed for EmbryoScope on embryos with known outcome, compared to standard morphology in a retrospective single center study.

**Methods:**

Morphokinetic annotation of 768 embryos with known outcome between 2013 -2015; corresponding to 116 D3 fresh embryos, 80 D6 frozen blastocysts, and 572 D5 blastocysts, fresh or frozen. The embryos were ranked by the KIDScore into five classes, KID1-5, and grouped into four classes based on standard morphology. Pregnancy rates, clinical pregnancy rates and live birth rates were compared. Combinations of morphology and morphokinetics were evaluated for implantation rates and live births.

**Results:**

Live birth rate increased with increasing KIDScore, from 19% for KID1 to 42% for KID5. Of all live births, KID5 contributed with 71%, KID4 with 20%, KID3 with 4%, KID2 with 4%, and KID1 with 2%. For morphology, the corresponding figure was 43% for Top Quality, 47% for Good Quality, 4% for Poor Quality, and 5% for Slow embryos. For day 3 embryos, KID5 embryos had the highest live birth rates, and contributed to 83% of the live births; whereas the second best morphological class had the highest live birth rate and contributed to most of the live births. For blastocysts, the KIDScore and morphology performed equally well. Combining morphology and morphokinetics indicated stronger predictive power for morphokinetics.

**Conclusions:**

Overall, the KIDScore correlates with both implantation and live birth in our clinical setting. Compared to morphology, the KIDScore was superior for day 3 embryos, and equally good for blastocysts at predicting live births.

## INTRODUCTION

The successful culture, evaluation and selection of embryos often determine the outcome of infertility treatment. The desire to reduce multiple births by a single embryo transfer has increased the burden on the clinical embryologist to choose the embryo with the highest ability to give the patient a healthy baby from a cohort of embryos. Traditionally, the evaluation and selection of embryos is done using morphology, often with daily removals and evaluations outside the safer environment of the incubator. The use of time-lapse incubators in clinical IVF laboratories has given embryologists access to thousands of multifocal images of each embryo. It provides a solution to the 'observational dilemma' that the likelihood of selecting the right embryo increases with the number of observations, but every observation poses a threat to the embryo as it gets exposed to sub-optimal culture conditions (Pribenszky *et al*., 2017). More information gathered about an embryo's development makes it fair to assume that more assumptions can be made regarding the embryo's implantation ability.

Time-lapse systems have been in clinical use in nearly a decade. The use of time-lapse images to annotate cleavage times and patterns is called morphokinetics. Each event in the development of the embryos can be measured often as hours post insemination (HPI), and it is referred to as a parameter. Events in embryo development have been linked to blastulation, implantation and chromosomal content. (Wong *et al.,* 2010; Meseguer *et al*., 2011; 2012; Azzarello *et al*., 2012; Cruz *et al*., 2012; 2013; Dal Canto *et al*., 2012; Rubio *et al*., 2012; Chamayou *et al*., 2013; Campbell *et al*., 2013; Liu *et al*., 2014; Hlinka *et al*., 2012).

By combining several parameters, algorithms, selection or de-selection models have been developed. These models are often developed from relatively small data sets, and/or clinical/chain specific data sets. Since morphokinetics is influenced by a number of external factors, the optimal time range for a high quality embryo may differ from clinic to clinic (Wale & Gardner, 2010; 2016; Ciray *et al*., 2012; Dal Canto *et al*., 2012; Kirkegaard *et al*., 2013; Muñoz *et al.,* 2012; 2013).

Meseguer *et al.* (2011) published a hierarchical model. First, a morphological screening excludes arrested or degenerated embryos, giving them an embryo score F. Secondly, embryos possessing exclusion criteria are given an embryo score E (uneven blastomere size at the two cell stage, multinucleation at the four cell stage, or abrupt division from 1 to 3 or more cells). Then, the morphokinetic absolute parameter t5, and the relative parameters s2 (t4-t3) and cc2 (t3-t2) are used to rank the remaining embryos. In total, ten embryo classes are created, which correlates with implantation ability. They later validated the model in a multicenter setting within the same IVF concern (Meseguer *et al*., 2012; Rubio *et al*., 2014). However, three external validations have failed to repeat the findings (Best *et al.,* 2013; Yalçinkaya *et al*., 2014; Fréour *et al*., 2015). In a retrospective analysis prior to this study, we investigated the potential of the Meseguer model in our clinic. Due to the inclusion of morphological parameters with higher subjectivity and lower intra-observer agreement, we failed to reproduce the predictive power of this selection model (Adolfsson & Nowosad, submitted manuscript).

Conaghan *et al.* (2013) published a computer-automated blastocyst prediction model, named the Eeva™ Test. The model uses two early cleavage intervals; t3-t2, ideal period 9.33-11.45 HPI, and t4-t3, ideal period 0-0.73 HPI. Embryos inside the ideal periods have a high likelihood of forming a clinically usable blastocyst, and embryos outside the ideal periods have a low likelihood. Kirkegaard *et al.* (2014) externally validated this model in a retrospective study with implantation as endpoint. Implantation rates were higher in the high ranked embryo subpopulation compared to the whole cohort. However, 50.6% of the embryos that implanted were ranked as unusable, and a strict usage of the model would have resulted in discarding of those embryos. The authors proposed the strict time frames as a likely explanation for the low model specificity, when applied to another clinic. Adamson *et al*. (2016) tested the same model in combination with morphology in a prospective concurrent-controlled study. The test group had embryo transfer based on both morphology and time-lapse data using the Eeva™ model, whereas the control group had embryo selection and transfer solely based on morphology. Implantation and clinical pregnancy rates were significantly higher in the test group.

In 2014, an updated version of the Eeva™ model was released, adding a third category of embryos using the same time-lapse parameters (VerMilyea *et al*., 2014). The model was validated by the developers in a multicenter retrospective study, with higher implantation rates in the High and Medium groups, compared to the Low group. External validation in the form of a prospective two-center pilot study failed to improve the outcome when combining morphology and Eeva™ model (Kieslinger *et al*., 2016).

In 2015, the Meseguer team published a version of their model (Basile *et al*., 2015), where the s2 parameter was removed, and the embryos were ranked into four categories based on t2-t3 interval, t3 and t5 ranges. The model was validated on a different subgroup of patients and the embryo ranks showed to correlate to implantation rates. They also correlated the same model to chromosomal content (Basile *et al*., 2014). Liu *et al*. (2016) published a deselection model in 2016, combining morphological features on day 2 with morphokinetic parameters (t8, s2, t5 in relation to tPNf). Their model ranked embryos from A+F with corresponding decreasing implantation rates. Milewski *et al*. (2015) ranked the embryos into four categories based on t2 to t5, as well as intervals between these time points. In their publication, the embryo classes were correlated to blastulation as primary endpoint to our knowledge, these models have not been externally validated yet.

**Figure 1:**
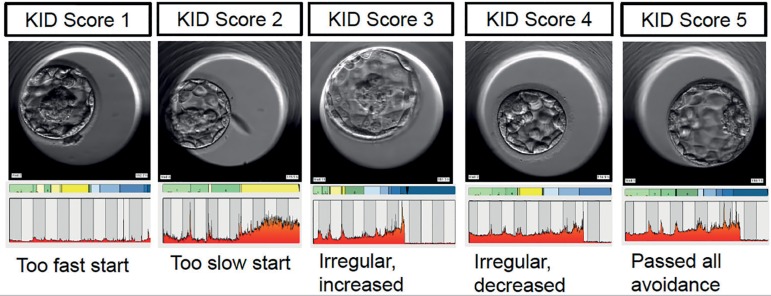
Examples of KID1 to KID 5 embryos from Örebro Fertility Clinic, with corresponding bar showing time-lapse annotation. KID1 embryos have a too fast start up to three cells. KID2 embryos have too slow initial development. KID3 embryos have irregular divisions with increasing development speed between the two and five-cell stages. KID4 embryos also have irregular divisions but with decreasing development speed between the two and five-cell stages, or, have not reached eight cells prior to 66 hours post insemination. KID5 embryos have passed all avoidance criteria. The perfect embryo should spend as little time as possible in yellow zones representing uneven cell numbers, and develop in a timely manner from one cell to two cells, from two cells to four cells, and so on. These embryos show that morphology is separate from morphokinetics. In each KID class, there are embryos with the potential to develop into clinically usable blastocysts that appear to be of high quality to the embryologist using standard morphology as embryo evaluation tool.

With the aim of introducing time-lapse as an embryo evaluation tool, we decided to validate the Embryo Scope's built-in algorithm named KIDScore D3 Basic. The KIDScore claims to be universal and applicable to all clinics. The model was constructed using a selection of 3275 transferred day-3 embryos with known implantation data (KID), of which ~800 implanted and ~250 yielded live births. The time-lapse data was gathered from a large database from 24 clinics, including both IVF and ICSI treatments, with embryo culture in both reduced and ambient oxygen levels (Petersen *et al.,* 2016). It is an avoidance model, which utilizes tPNf, t2, t3, t4, t5 and t8 to rank embryos into five morphokinetic classes: 1-5. The score from 1-5 is a relative measure of the embryo's implantation potential. In a first step, embryos with too fast initial development (t3-tPNf <11.48 HPI) are excluded as KID1. Next, embryos with too slow initial development (t3 ≥42.91 HPI) are excluded as KID2. An equation is added (t5-t3/t5-t2), which describes irregularities in the division pattern between the two-cell stage and the five-cell stage. This equation is used twice, first deselection embryos with an index <0.3408 as KID3, and then deselection embryos with an index of ≥ 0.5781 as KID4. In the last step, embryos which did not reach the eight-cell stage before 66 HPI are deselected as KID4. Hence, there are two types of embryos in KID4. All other embryos, i.e. embryos which have passed all avoidance criteria are ranked as KID5. See Figure 1 for examples of KID1-5 embryos. In their publication, describing the development of the algorithm, an implantation predictability of AUC 0.650 and a blastulation predictive power of AUC 0.745 when applied to day-3 embryos is reported. It is designed to keep many embryos in the highest ranks by a conservative approach, in contrast to a selection model with a narrower time range, with fewer embryos in the highest ranks.

In their publication, they compared the KIDScore to several other models for the endpoints: blastulation and blastocyst quality. As a reference, they used the morphological scoring system proposed by ASRM/ESHRE (Alpha Scientists in Reproductive Medicine & ESHRE Special Interest Group of Embryology, 2011). Only the KIDScore and the Liu's model surpassed the predictive power of morphology. However, their endpoint was subordinate to the strongest endpoint in IVF, i.e. the live birth rate. The use of time-lapse for all patients since 2012, in combination with single embryo transfer and rigid patient follow up, has provided us with a large database of embryos with morphokinetics, morphology and known implantation data. Prior to changing the embryo selection tool from morphology to morphokinetics, validation needs to take place to ensure quality control, high discrimination, objectively, and equal or better performance. The aim of this study was to validate the KIDScore in a clinical setting with an unselected population, in comparison to standard morphology scoring, with the primary endpoint of live births.

## MATERIALS AND METHODS

### Assisted reproduction treatment

We carried out this retrospective study on all patients between 2013 and 2015, at the Fertility Unit at Örebro University Hospital, Sweden. The clinic is a 100% time-lapse-clinic, culturing all embryos in EmbryoScope since 2012. All our subjects signed a written informed content. The local ethics committee (Regionala etikprövningsnämnden Uppsala, ethical approval Ö44-14) approved the study. Our only exclusion criteria was the patient's lack of consent.

All patients had controlled ovarian stimulation with either antagonist or agonist protocol. We adjusted the starting dose to female age, ovarian reserve and outcome of any previous cycles. When at least 3 follicles reached 18 mm, triggering was performed using recombinant human chorionic gonadotropin. Oocytes were retrieved 36 hours after triggering. The oocytes were fertilized by standard gamete co-incubation (referred to as IVF) or ICSI. IVF oocytes were cultured overnight in a standard incubator (+37ºC, 6% CO_2_) before cumulus cells were removed and all oocytes placed in the EmbryoScope. ICSI oocytes were placed in EmbryoScope (+37ºC, 6% CO_2_, 5% O_2_) directly after microinjection. Embryos were cultured in sequential media, with half media change done in the afternoon of days 2 and 4.

Transfer of a single fresh embryo was done on either day 3 or day 5, depending on medical history, day of oocyte pick-up and number of available embryos. Selection of embryo for transfer was done solely on morphologic criteria, using the time-lapse images instead of traditional microscopy. We used the Gardner Schoolcraft criteria (Gardner *et al*., 2000) to score blastocysts at approximately 116 HPI. Local laboratory criteria based on number of blastomeres, degree of fragmentation and evenness of blastomeres was used for cleavage stage embryos at approximately 68 HPI. Surplus embryos were cultured to blastocyst stage and vitrified when reaching clinical usage criteria (grade 3BB or better). If patient returned for a frozen thaw replacement cycle, the best available embryo was thawed approximately 2 hours prior to transfer. Survival and re-expansion of collapsed blastocysts was done prior to frozen single embryo transfer, and only embryos with full survival and at least 80% re-expansion were transferred. All frozen-thaw cycles was done unstimulated.

A home urine pregnancy test was taken 16 days after embryo transfer. If pregnant, an early vaginal ultrasound was performed in week 6 of gestation to confirm viable pregnancy and number of sacs and fetuses. Outcome of treatment (live-born baby) was obtained from all participating patients through follow-up questionnaires and/or phone calls.

### KID Score Evaluation

Time-lapse annotation was performed in retrospect on all embryos transferred fresh/frozen between 2013-2015. 768 transferred embryos were included (IVF n=342, ICSI n=426). The cohort consisted of 116 D3 fresh transferred embryos, 80 D6 vitrified/warmed transferred embryos, and 572 D5 blastocysts (287 fresh and 287 frozen). The following parameters were annotated for each embryos; tPNa as the time of appearance of pronuclei, tPNf as the time of fading of pronuclei. t2, t3, t4, t5, t6, t7, t8, t9+ was defined as the times for the corresponding number of cells. tM was defined as the first frame of the morula stage, tSB as the first frame with presence of blastocoel, tB as the first frame of a fully formed blastocyst, tEB as the first frame showing expansion of the zona pellucida with enlargement in size. The 'Compare and Select' feature in EmbryoViewer software (Vitrolife, Denmark) with KIDScore D3 Basic was used to rank embryos into KID 1-5.

### Statistics

For statistical purposes, the embryos were categorized into morphological classes based on their grade. The blastocysts were classified as belonging to the Top Quality Embryo (TQE) Group; blastocysts with an A for ICM and/or TD, to the Good Quality Embryo (GQE) Group; blastocysts with B for both ICM and TD, to the Poor Quality Embryo (PQE) Group; blastocysts with a C for ICM and/or TD, or Slow; embryos with an expansion grade of 0, 1 or 2 (pre-blastocyst stage embryos). Day-3 embryos were categorized into three classes based on their morphological evaluation. Day-3 embryos with exactly 8 cells, less than 20% fragmentation and even blastomeres were classified as TQE. Day-3 embryos with 6-10 blastomeres or with more than 20% fragmentation, or uneven cells were classified as GQE. Day-3 embryos with less than 6 cells, or more than 10 cells, and/or <50% fragmentation were classified as PQE.

Pregnancy rate (PR) was calculated as the percentage of transfers leading to a rise in beta-HCG. Clinical pregnancy rate (CPR) was calculated as percentage of transfers leading to intrauterine gestational sacs with fetal heartbeat observed by transvaginal ultrasonography. Live birth rate (LBR) was calculated as the percentage of live born babies. PR, CPR, LBR were calculated and compared for each morphokinetic score and for each morphological score, with significance testing using fishers exact t-test.

## RESULTS

The transfer of 768 embryos resulted in 380 positive pregnancy tests (PR 49.5%), 299 ongoing pregnancies as detected by early ultrasound (CPR 38.9%) and 283 live births (LBR 36.8%). There was no significant difference in outcome between IVF and ICSI (LBR for ICSI 37.8%, for IVF 36.1%). Blastocyst transfers resulted in higher LBR compared to cleavage stage embryos, and transferring D5 blastocysts resulted in higher LBR compared to transferring D6 blastocysts. See Table 1 for details.

**Table 1:** Embryos included in the study. The cohort consisted of cleavage-stage embryos, transferred fresh on day 3 of development, and blastocysts, either day-5 or day-6, transferred fresh or vitrified/warmed. Pregnancy rates (PR), clinical pregnancy rates (CPR) and live birth rates (LBR) are expressed in percentages (%) and in absolute numbers (n). LBR for day 3 embryos were lower compared to blastocysts (*p*=0.034) and D6 transfers lower than D5 transfers (*p*=0.04)

Day of transfer	Type	Embryos (n)	PR % (n)	CPR % (n)	LBR % (n)
**D3 **	Fresh	116	31.0 (36)	28.4 (33)	25.0 (29)
**D5 **	Fresh	287	55.7 (160)	44.9 (129)	43.2 (124)
**D5 **	Frozen	285	54.4 (155)	38.9 (111)	36.5 (104)
**D6 **	Frozen	80	35.0 (28)	32.5 (26)	32.5 (26)
	**Total**	**768**	**49.5 (380)**	**38.9 (299)**	**36.8 (283)**

### Embryo evaluation using the KIDScore

**Figure 2:**
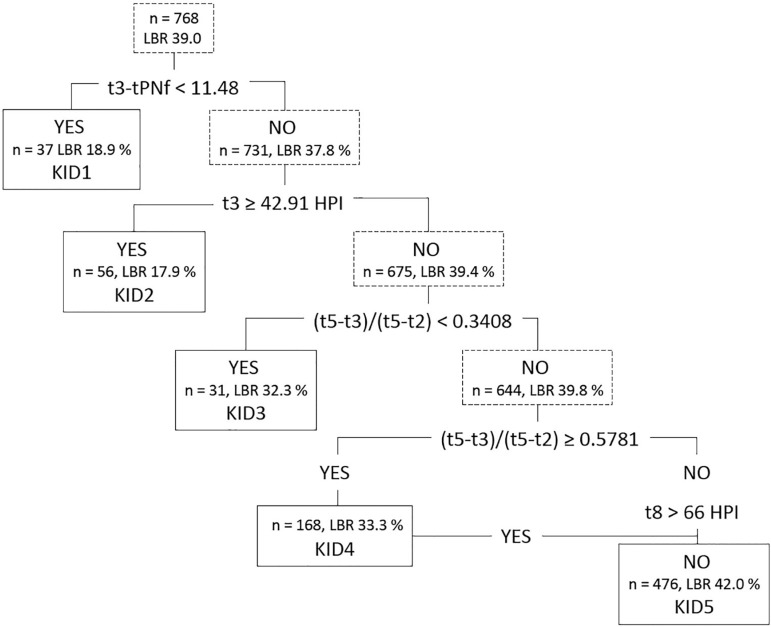
The hierarchical model with five splits used to generate the KIDScore D3 Basic, adapted from Petersen *et al.*, 2016. Number of inputted embryos, distribution of embryos into KIDScore classes, and corresponding live birth rates (LBR) are shown in the figure. The embryos are ranked based on i) initial cleavage speed up to three cells, ii) t3 time point, ,iii and iiii) irregular cell divisions from 2 cells to 5 cells as described by the (t5-t3)/(t5-t2) equation, and iiiii) on reaching eight cells before 66 hours post insemination (HPI). KID4 embryos are composed of two types of embryos: those that have irregular divisions and those that do not reach eight cells prior to 66 HPI.

The algorithm used to score all embryos was accessed through the EmbryoScope software (Figure 2). Ranking all embryos using KIDScore resulted in an uneven distribution into five classes. The majority of embryos belonged to KID5 (62%), followed by KID4 (22%), KID3 (4%), KID2 (7%) and KID1 (5%).

**Figure 3:**
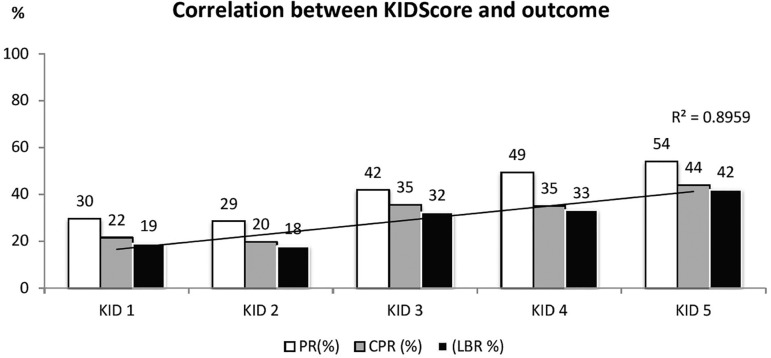
Correlation between KIDScore and outcome, all embryos included. Highest live birth rate (LBR) was found with KID5, followed by KID4, KID3, and then similar values for KID1 and KID2. Pregnancy rates (PR) and clinical pregnancy rate (CPR) show the same pattern. A regression curve with correlation coefficient is presented for LBR.

**Figure 4:**
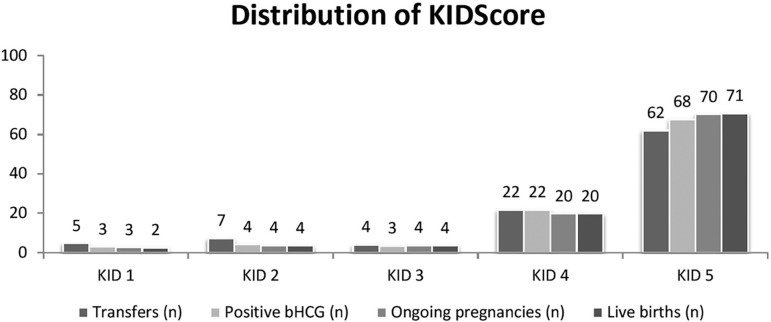
Distribution of the KIDScore - all embryos included. The figure shows the percentage of each KIDScore class on different categories (number of transfers, number of positive pregnancy tests, number of ongoing pregnancies, and number of live births).

Looking at all embryos, the LBR increased from 18.9% for KID1, 17.9% for KID2, 32.3% for KID3, 33.3% for KID4 to 42.0% for KID5. The distribution of the KIDScore classes on transferred embryos, positive pregnancy tests, ongoing pregnancies and live births show a prevalence of KID5 embryos in all categories. Of all live births, 71% originated from a KID5 embryo, 20% from KID4, and only 9% from the remaining KID1-2-3. See Figures 3 and 4 for details.

When splitting embryos based on day of transfer, the distribution of embryos into KIDScore classes, and their corresponding PR, CPR and LBR showed similar patterns as for all embryos combined. For blastocysts, LBR increased from 21.2% for KID1 to 43.8% for KID5. For cleavage-stage embryos, LBR increased from 0% for KID1-2, to 33.3% for KID5. Of all live births after day-3 transfers, KID5 embryos accounted for 83%. See Table 2.

**Table 2:** Morphokinetics and outcome, as well as the KIDScore contribution, split by day of transfer. Day-5 and day-6 blastocysts are grouped together. For each KIDScore, the data is presented as PR, CPR and LBR (in bold). For each category, the contribution of each KIDScore class is presented as percentage (in italics)

	DAY 3 EMBRYOS								BLASTOCYSTS							
CLASS	N	C (%)	PR	C (%)	CPR	C (%)	LBR	C (%)	n	C (%)	PR	C (%)	CPR	C (%)	LBR	C (%)
**KID1**	4	*3.4*	**25.0**	*2.8*	**25.0**	*3.0*	**0.0**	*0.0*	33	*5.1*	**30.3**	*2.9*	**21.2**	*2.6*	**21.2**	*2.8*
**KID2**	11	*9.5*	**9.1**	*2.8*	**9.1**	*3.0*	**0.0**	*0.0*	45	*6.9*	**33.3**	*4.4*	**22.2**	*3.8*	**22.2**	*3.9*
**KID3**	7	*6.0*	**28.6**	*5.6*	**28.6**	*6.1*	**14.3**	*3.4*	24	*3.7*	**45.8**	*3.2*	**37.5**	*3.4*	**33.3**	*3.1*
**KID4**	22	*19.0*	**22.7**	*13.9*	**22.7**	*15.2*	**18.2**	*13.8*	146	*22.4*	**54.1**	*23.0*	**37.7**	*20.7*	**35.6**	*20.5*
**KID5**	72	*62.1*	**37.5**	*75.0*	**33.3**	*72.7*	**33.3**	*82.8*	404	*62.0*	**56.7**	*66.6*	**45.8**	*69.5*	**43.8**	*69.7*

N = number of embryos in each KIDScore class, PR = pregnancy rate, CPR = clinical pregnancy rate, LBR = live birth rate, C = contribution.

### Evaluation of embryos using morphology

**Figure 5:**
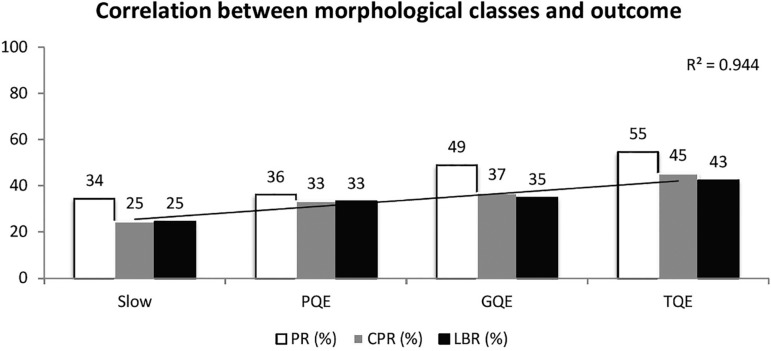
Correlation between morphological classes and outcome, for all embryos included in the study. The LBR increases from 25% for PQE, to 43% for TQE. PQE = poor quality embryos, GQE = good quality embryos, TQE = top quality embryos, PR = pregnancy rate, CPR = clinical pregnancy rate, LBR = live birth rate. A regression curve with correlation coefficient is presented for LBR.

**Figure 6:**
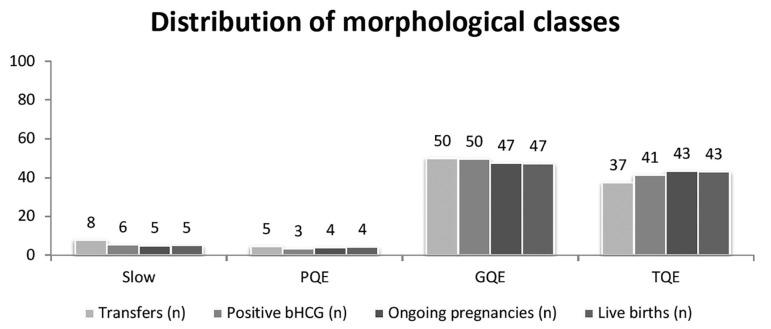
Distribution of morphological classes, all embryos included in the study. The figure shows the percentage of each morphological class on each category (number of transfers, number of positive pregnancy tests, number of ongoing pregnancies, and number of live births). Surprisingly, the majority of embryos are ranked as second highest class GQE, not in the best class, with very few embryos in the poorest categories. PQE = poor quality embryo, GQE = good quality embryo, TQE = top quality embryo.

The blastocysts were categorized into four classes- TQE, GQE, PQE and Slow-, whereas cleavage-stage embryos were categorized into only three classes - TQE, GQE, PQE. The embryo distribution was uneven, with TQE accounting for 37% of the total embryos, GQE for 50%, PQE for 5% and Slow 8%, respectively. Looking at all embryos, LBR decreased from 43% for TQE, 35% for GQE, 33% for PQE, and 25% for Slow. Due to the distribution of embryos, the majority of live born babies are attributed to the second highest category 'GQE' (47%) followed by TQE (43%) and only 4% from PQE and 5% from Slow embryos. See Figures 5 and 6.

When splitting the embryos based on day of transfer, a different pattern is found for cleavage-stage embryos. Of the 116 day-3 embryos, 61% were assigned as GQE, 29% as TQE and 10% as PQE. LBR was 18% for PQE, and 32% for both TQE and GQE, i.e. morphology could not identify embryos with the highest ability to result in live birth from day-3 morphological data. The skewed distribution with the vast majority of embryos assigned as GQE resulted in 66% of live births from day-3 transfers originating from the second-best morphology class, more than double that of TQE (28%). For blastocysts, LBR was similar for PQE and GQE, and significantly higher in TQE (*p*=0.039). However, due to the distribution with more embryos assigned to GQE, the contributions of TQE and GQE on live births were identical (45.3%). See Table 3.

**Table 3:** Morphology and outcome, as well as distribution of embryos, split by day of transfer. Day-5 and day-6 blastocysts are grouped together. For each morphological class, the data is presented as PR, CPR and LBR (in bold). For each category, the contribution of each KIDScore class is presented as percentage (in italics)

	Day 3 embryos								Blastocysts							
CLASS	n	C (%)	PR	C (%)	CPR	C (%)	LBR	C (%)	n	C (%)	PR	C (%)	CPR	C (%)	LBR	C (%)
**Slow**	-	-	-	-	-	-	-	**-**	61	*9.4*	**9.4**	*6.1*	**24.6**	*5.7*	**24.6**	*5.9*
**PQE**	11	*9.5*	**18.2**	*5.6*	**18.2**	*6.1*	**18.2**	*6.9*	25	*3.8*	**3.8**	*3.2*	**40.0**	*3.8*	**36.0**	*3.5*
**GQE**	71	*61.2*	**32.4**	*63.9*	**31.0**	*66.7*	**26.8**	*65.5*	31	*48.0*	**48.0**	*48.3*	**38.3**	*45.3*	**36.7**	*45.3*
**TQE**	34	*29.3*	**32.4**	*30.6*	**26.5**	*27.3*	**23.5**	*27.6*	23	*38.8*	**38.8**	*42.4*	**47.8**	*45.7*	**45.5**	*45.3*

n = number of embryos in each KIDScore class, PR = pregnancy rate, CPR = clinical pregnancy rate, LBR = live birth rate. PQE = poor quality embryo, GQE = good quality embryo, TQE = top quality embryo, C = contribution.

## DISCUSSION

The KIDScore is an implantation model designed for EmbryoScope, to aid in the selection of viable embryos. The model is developed on data sets from many clinics, and therefore supposedly applicable to any clinic. This is, to our knowledge, the first study to externally validate this model, and our results support the ability of the KIDScore to identify high performing embryos.

In their original study, Petersen *et al*. (2016) further validate the KIDScore on its power to predict blastocyst formation. They applied the algorithm on ~11,000 normally fertilized embryos and found an association between the KIDScore and the proportion of blastocysts, as well as the quality of formed blastocysts. Although not intended as a blastocyst prediction model, the authors state that it can be used as such. Therefore, we applied the KIDScore to 656 normally fertilized oocytes between January and April of 2015 with the endpoint of clinically usable blastocyst, i.e. grade 3BB or better, on day 5 of development. Correlation was found between usable blastocyst development and the KIDScore. The blastocyst formation rate was 66% for KID5, 36% for KID4, 21% for KID3, 21% for KID2 and 23% for KID1. Because blastulation is a prerequisite for implantation, this finding is not surprising for an implantation model. It adds a feature that could help the clinical embryologist when deciding, on day 3, whether to continue to culture or transfer at that stage, since all information needed to score the embryos is available after 66 hours of culture.

In the original paper, the algorithm was designed for day-3 transfers only. They reported implantation rates of 36% for KID5, 23% for KID4, and 11% for KID 1-2-3 combined. Our corresponding numbers are 33%, 23% and 18%. Hence, we reproduced the implantation selection ability of KIDScore for day-3 embryos. We also included blastocysts with the same results; increasing implantation rates for high KIDScores. We therefore show that the KIDScore predicts implantation for blastocysts in our clinical setting.

Besides validating the KIDScore as an implantation model, we used the strongest endpoint in IVF; live births. Applied to the whole cohort, KID5 embryos had a 2.2 fold likelihood of resulting in a live birth compared to KID1. For both cleavage stage embryos and blastocysts, the KIDScore functioned as a live birth predictor in our study.

In comparison to morphokinetics, our present embryo evaluation method had a poor correlation to both implantation and live birth on day 3. Embryos ranked as top quality based on morphology had a lower LBR compared to embryos ranked as good quality. This highlights the complexity and difficulty of scoring embryos on day 3, with rapid changes in appearance, formation and re-absorption of fragments. With morphokinetic, selection models in general, and with the KIDScore specifically, only a few objective parameters are used to rank the embryos (tPNf, t2, t3, t5, and t8). Prior to this validation, we performed a comparison between morphology and morphokinetics in terms of inter-observer and intra-observer agreement. For morphokinetics, all investigated parameters had a high agreement rate between observers and between repeated measurements. When evaluating an embryo, the outcome is independent of which embryologist annotates and when. All parameters included in the KIDScore model had an 'almost perfect agreement' except t8, which had 'strong agreement'. The notion that the objectivity and reproducibility of morphokinetics adds value to the more subjective method of morphology, especially on day 3, is strengthened from the findings from Adamson *et al*. (2016) by selecting embryos evaluated by morphokinetics together with morphology, in comparison to morphology alone, they were able to improve pregnancy rates. Embryos ranked as high quality based on time-lapse had higher implantation rates (45% compared to 20%).

In contrast, morphological evaluation was capable of identifying blastocysts with high LBR. The embryos ranked as top quality had the highest LBR - 45.5%, but because GQE was the most common score, both TQE and GQE contributed equally to live births - 45%. Using morphokinetics, the highest KID5 score had a LBR of 44% but contributed with 70% of the live births, due to more embryos contained in the highest category. This is a feature of the KIDScore and a feature of deselection models in general. It reduces the risk of scoring an embryo with a fair chance of resulting in a successful outcome as poor.

Although a high morphokinetic score often accompanies a high morphological score, it is not always the case. Embryos with irregular division patterns, like direct cleavage, can develop into morphologically excellent blastocysts. However, their implantation ability is substantially reduced (Rubio *et al.,* 2012; Zaninovic *et al*., 2013). Given that, we only transfer one embryo at a time, avoiding these embryos that are tempting to the eye but unlikely to give the patient a healthy baby, will save costs and shortens the time to pregnancy. Indeed, looking at the top quality embryos shows that KID5 is the most frequent score, but all morphokinetic classes are represented. LBR is reduced from 45% if it is a TQE/ KID5, to 17%, if it is a TQE/KID1. On the contrary, for a KID5-embryo, the LBR will not drop equally low when reducing morphological classes (KID5/TQE 45%, KID5/GQE 41%, KID5/PQE 40%, and KID5/Slow 34%). Thus, morphokinetics appears to have a bigger impact on LBR in this study.

There are limitations to this study. First, despite including almost 800 embryos, the uneven distribution of embryos makes some categories small, hence limiting statistical power. The study was retrospective and presumably, the best available embryo in each IVF cycle was selected on basis of morphology. Grouping embryos based on morphological features was necessary to reduce the number of categories, but it might mask important correlations between embryo quality and ability.

**Figure 7:**
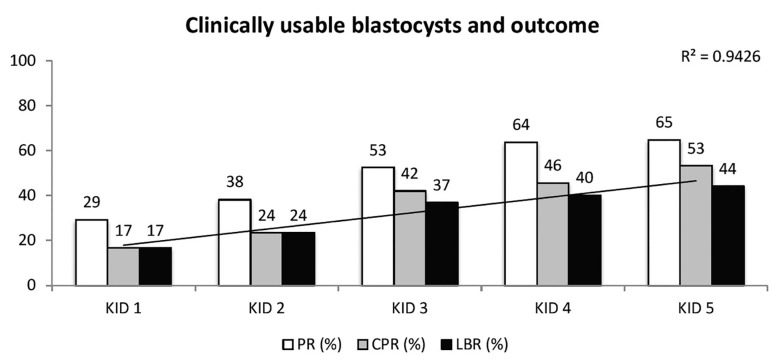
Correlation between clinically usable blastocysts, i.e. Grade 3BB or better (Gardner Schoolcraft criteria) and outcome. Highest LBR is found for KID5 blastocysts, a 2.6 increase compared to KID1. PR = pregnancy rate, CPR = clinical pregnancy rate, LBR = live birth rate. Regression curve with correlation coefficient is presented for LBR

Overall, our study shows that the KIDScore in our settings is capable of selecting embryos with the highest ability to give the infertile patient a live birth, regardless of type of embryo or length of culture. For cleavage-stage embryos, the KIDScore was significantly better of predicting live births. For blastocysts, the strongest use of KIDScore might be in combination with morphology. Blastocysts scored as 'clinically usable', i.e. Grade 3BB or better, had higher chances to result in live birth if being KID5 (Figure 7). A proposed way of working could be to culture embryos to day 3 and perform KIDScore annotation for all embryos that are not clearly arrested or degenerated. If several embryos receive high KIDScore rates, the culture can be prolonged with the reassurance of high likelihood of reaching blastocyst stage as clinically useful blastocysts. On day 5, the KIDScore can be used to choose the embryo with the highest ability to result in a live birth among the cohort of blastocysts deemed as clinically usable by the laboratory standard operating procedure.

The KIDScore share time-lapse parameters with many of the other morphokinetic models. Most often, those models are based on a relatively small number of embryos in a single center setting, and although those models fit the original set of data well, they have failed independent validation by others. In contrast, the KIDScore was developed from 24 data sets, all from clinics doing assisted reproduction in slightly different ways. We applied it retrospectively to all transferred embryos from 2013 to 2015, regardless of day of transfer (day 3 or blastocyst), type of cycle (fresh or frozen), or type of treatment (IVF or ICSI). Here we show that is functions as a blastocyst prediction algorithm, as implantation prediction algorithm, and as live birth predictor in a clinical setting using sequential media, reduced oxygen, both IVF and ICSI treatments and both antagonist and agonist type of stimulation. The differences found for IVF and ICSI morphokinetics (Cruz *et al*., 2013; Liu *et al*., 2015; Kirkegaard *et al*., 2016) is caused by differences in early cleavage times. These cleavage times are related to time of insemination, but it is difficult to establish such time for IVF. The time of gamete co-incubation is often used, although fertilization may take place much later. By using tPNf as a starting point, the differences between IVF and ICSI is removed. In KIDScore, t2, t3, and t5 are used in relation to each other and are therefore independent of time of insemination. t3 is used twice, and in one of the splits, t3 is used in relation to tPNf. The uncertainty of time of insemination for IVF is almost excluded from the model. No differences were found between IVF-derived embryos compared to ICSI-derived embryos in this validation in terms of embryo distribution or LBR (data not shown).

The clinical use of time-lapse incubators with regards to improved culture conditions and increased ease of quality control makes them invaluable in clinical practice - even without using morphokinetics for embryo evaluation and selection. A recent meta-analysis on morphokinetics showed increased pregnancy rates, reduction in early pregnancy loss and increase in live births when selecting embryos using the morphokinetic embryo evaluation (Pribenszky *et al*., 2017). This study supports that when using the information obtained by time-lapse, the likelihood of choosing the right embryo increases.
